# Primary care pediatricians’ involvement in influenza vaccination campaign in Italy

**DOI:** 10.1186/s13052-025-02093-6

**Published:** 2025-08-22

**Authors:** Rosaria Indaco, Francesca Leoni, Costantino Panza, Paolo Giorgi Rossi

**Affiliations:** 1 PCPs of Azienda USL-IRCCS di Reggio Emilia, Reggio Emilia, Italy; 2 Epidemiology Unit, Azienda USL-IRCCS di Reggio Emilia, via Amendola 2, Reggio Emilia, 42122 Italy

## Abstract

**Background:**

In 2022, the Italian Ministry of Health extended free annual influenza vaccination to all children aged 6 months to 6 years. Since coverage remained low, the Emilia-Romagna region authorized primary care pediatricians (PCPs) to vaccinate both healthy and chronically ill children in 2023, with the aim of increasing vaccine uptake. This study aims to investigate factors influencing PCPs’ participation in the 2023/24 influenza vaccination campaign in Emilia-Romagna, including perceived barriers and satisfaction among participant pediatricians.

**Methods:**

An anonymous online survey was distributed between January 13–28, 2024, to all PCPs in Emilia-Romagna (*N* = 557), to evaluate participation in the 2023/24 pediatric influenza vaccination campaign. The survey explored demographic and professional information, vaccination practices and training, and perceived campaign impact and satisfaction. Outcome variables included campaign participation, perceived problems and satisfaction levels. Logistic regression models to assess determinants of participation and Cochran-Armitage test to explore trends in satisfaction levels were used.

**Results:**

319 PCPs (57.2% of total) responded to the survey, of which 223 (70.8%) joined the campaign. The number of patients in care, working in group clinic vs. single PCP clinic (OR 2.45), experience as vaccinator (OR 3.12) and PCP’s anti-flu vaccination (OR 1.94) were positively associated with participation; age, training at post-graduate school (OR 0.54) and self-reported competence in vaccination (OR 0.62) were negatively associated with participation. Among non-participants, only 25% reported at least one difficulty, mainly lack of fridge or emergency kit and fear of managing adverse reactions; among participants, 85% reported at least one problem, mostly related to limited communication campaigns, organization and fear of managing adverse reactions. Satisfaction levels were higher among male PCPs, those with experience as vaccinators, who vaccinated more children, and those with shorter administration times. The main reasons for dissatisfaction were related to the organization time, and lack of information for parents and economic advantage.

**Conclusions:**

This study highlights the barriers that must be removed to achieve effective PCPs’ involvement in the vaccination campaign. Future strategies should focus on strengthening logistical support, tailored training and parent communication, and promoting shared models of care to enhance effectiveness and sustainability of vaccination programs.

## Background

Many health organizations recommend flu vaccination for children in their first 5–6 years of life, as they are at higher risk of severe illness or complications when infected. The vaccine also helps reduce the circulation of the influenza virus among adults and the elderly [1, 2, 3].

Parental views and practices surrounding childhood vaccination are shaped by several factors, including access to vaccination services and experiences with healthcare providers [4]. Significantly more parents chose to immunize their children after consulting with trained physicians [5]; a familiar environment also fosters trust from parents [6]. Thus, the success of the vaccination campaign largely depends on how well pediatricians inform families and how early they provide this information, which can increase adherence, reduce concerns about side effects, and ensure ongoing vaccination, particularly for children with chronic conditions [7, 8]. Logistical barriers are reported as an important reason for missed or delayed immunizations [9, 10]. Offering the flu vaccine in the PCP’s office could increase adherence to vaccination recommendations [11]. The PCP can vaccinate the child at any point during the vaccination season, preventing delays that may increase the risk of illness and missed vaccinations, with flexible scheduling of vaccination visits and reducing the number of accesses [9]. PCPs can further reduce access barriers by offering walk-in clinics, dedicated vaccination stations, special vaccination hours, or administering the vaccine during routine visits for non-feverish illnesses [12].

Additional strategies to improve vaccination adherence include distributing informational posters, sending patient reminders, and encouraging colleagues to support vaccination campaigns [12, 13]. Face-to-face communication about childhood vaccination is common and typically focuses on acceptance and awareness. Healthcare providers, like PCP, who regularly engage in face-to-face discussions with parents, have a positive impact on vaccine acceptance [14].

However, barriers to vaccination also exist among pediatricians. Despite providing accurate vaccine education, 70% of pediatricians report suboptimal attitudes and confidence regarding vaccines, and only 63% receive the flu vaccine annually in Italy [15]. Low vaccination rates among healthcare professionals are often linked to insufficient knowledge of the vaccine’s value, concerns about its efficacy, fears of side effects, and a perceived low risk of contracting influenza [16, 17]. Some doctors hesitate to recommend the vaccine due to fears that it may cause illness or damage the immune system [18].

In Italy, until 2021, annual flu vaccination was only recommended for children with high-risk medical conditions. However, starting in 2022, the Ministry of Health recommended that all children aged 6 months to 6 years be vaccinated annually through a free immunization campaign [2]. Despite these recommendations, flu vaccination coverage among pediatric populations is very low. According to the Italian Ministry of Health’s 2022–2023 report, vaccination rates were 7.2% for children aged 6–23 months and 9.2% for children aged 2–4 years, with regional variations (3.4% in Emilia-Romagna and 10.5% in Tuscany) [19], mainly targeting children with chronic conditions.

In Italy, the regional Health Authority decide how to organize the flu vaccination, while the implementation is put in place by the Local Health Agencies (LHA). Pediatric vaccinations are in most cases directly provided by LHA via dedicated outpatient clinics. Nevertheless, some regions delegate this task to PCPs. Since 2023, the Emilia-Romagna region has decided to involve PCPs in the pediatric flu vaccination campaign authorizing all PCPs to administer vaccines to both healthy children aged 6 months to 6 years and chronically ill children aged 6 months to 16 years in their clinics [20]. Thus, PCPs have only recently begun universal flu vaccination campaigns and it was crucial to assess how the PCPs accepted this new task and how the campaign designed by the Emilia-Romagna Health Authority impacted on their activities and how it could be improved.

## Methods

### Aim of the study

This study aims to assess factors that may limit or encourage PCPs' participation in the vaccination campaign in Emilia-Romagna and explore ways to improve work organization and increase vaccination offers in pediatric offices. For this purpose, a survey was carried out with an online questionnaire proposed to all PCPs in the Emilia Romagna region.

Specifically, the objectives of the study are:

1. assessing the determinants of participation to the vaccination campaign;
2. investigating the problems noticed by PCPs in the campaign;
3. investigating the level of satisfaction among those who joined the campaign.

### Setting

The Emilia-Romagna Region is located in the Po valley, Northern Italy, it has about 4.2 million inhabitants and it is divided in 8 LHA. In Italy, PCPs are payed by the National Health System (NHS) with an exclusive agreement. In 2023, they could have a maximum of 880 children from 0 to 14 years. They can work alone in a single PCP clinic, in network, or in group hosted in private or public primary care centers.

The Regional Health Authority (RHA) organized the 2023/24 flu vaccination campaign allowing the voluntary participation of the PCP. The cost of the vaccination campaign was covered by RHA, distribution was borne by LHA. In the campaign 23/24, the LHAs procured and delivered 30 doses for each PCP (except in Ferrara and Piacenza where the number of available doses for PCPs was higher). The PCPs who adhere to the vaccination campaign, receive a compensation per dose established by the regional agreement, and increased by possible agreement with LHA, on specific objectives. The PCPs actively calls patients, vaccinates them and registers them on the regional vaccination registry.

### Survey design and ethical considerations

The survey was promoted by the Reggio Emilia PCPs and received the endorsement of the Reggio Emilia LHA and the PCP associations of all the region. Given the complete anonymization of the data collected and the observational design, the study did not require ethical approval in accordance with Italian law (Gazzetta Ufficiale no. 76, dated March 31, 2008).

PCPs from the Emilia Romagna region in Italy were contacted via email and invited to participate in an online survey conducted from January 13, 2024, to January 28, 2024.

The survey was distributed through Google Forms and was designed to be anonymous, with all responses being non-mandatory. The objective was to gather data on the first influenza vaccination campaign extended to the entire pediatric age group, to assess the factors influencing PCP’s adherence to the vaccination campaign and, through an analysis of the barriers and determinants of PCP’s satisfaction, to identify critical issues that could be addressed to improve participation in future vaccination campaigns.

To develop the questionnaire, we referenced similar studies, such as the Treatment Self-Regulation Questionnaire for Healthcare Professionals’ Motivation towards Flu Vaccination (TSRQ-Flu). This scale has been shown to provide valuable insights into the roots of vaccine hesitancy among healthcare professionals, helping managers design evidence-based interventions to promote vaccination among staff [21]. Additionally, we referred to studies that assessed health workers’ attitudes, behaviors, and knowledge regarding the flu vaccine, often using computerized questionnaires on platforms such as EUSurvey [22].

However, many questions in our questionnaire have been adapted to the role of PCP in Italy. Our survey assessed several factors to better understand organizational patterns and difficulties encountered.

### Survey structure

The questionnaire was organized into three parts, the first and the second parts targeted all PCPs, while the third one targeted only those who participate in the campaign:

1. Demographic and Professional Information: The first section gathered data on the respondent’s age, gender, province of practice, number of patients managed, type of work organization, level of knowledge regarding vaccinations, and whether they were designated as vaccinators.
2. Vaccination Practice and Training: The second collected information on the number of children vaccinated (both healthy and with chronic conditions), the setting in which they administered the vaccine, whether study staff assisted in the vaccination process, and any challenges encountered during the campaign.
3. Perceived Campaign Impact and Attitude: The third section assessed the participants’ satisfaction with the campaign, the time spent on vaccinations, the perceived utility of the campaign, and the likelihood of their future participation. Additionally, PCPs who did not participate in the campaign were able to express their dissatisfaction and highlight any barriers that prevented their involvement.

### Definition of outcomes

The outcome variables are:

1. participation in the vaccination campaign, dichotomous variable with 2 categories: yes and no;
2. presence of problems related to the vaccination campaign. Difficulties were grouped as follows:
  logistical problems: lack of refrigerator, lack of emergency kits, difficulty in obtaining doses, insufficient number of doses;
  problems related to adverse reactions: fear of adverse reactions, fear of managing adverse reactions;
  organizational problems: waiting room, organization, informed consent, vaccine administration, registration, information campaign, collaboration with Primary Care Doctors (PCD);
3. level of satisfaction among participants in the campaign. Increasing values were assigned from 1 (very dissatisfied) to 5 (very satisfied) to the ordered levels of the variable.

### Variables of interest

The variables from the survey are:

1. characteristics of pediatricians: age range, categorized as 4 groups (25–35, 36–45, 46–55, >56); sex (male or female); 23/24 PCP’s vaccination (yes or no);
2. training and experience: current or previous experience as Asl vaccinator (yes or no); training at post-graduate school (yes or no); self-reported competence as vaccinator (yes or no); refresh courses (yes or no);
3. characteristics of clinical practice: working province, categorized as 9 groups (Piacenza, Parma, Reggio Emilia, Modena, Bologna, Ferrara, Forlì-Cesena, Rimini, Ravenna); municipality residents, categorized as 3 groups (< 20000, 20000–50000, >50000); number of patients managed, categorized as 4 groups (< 500, 500–700, 700–900, >900); type of work organization, categorized as 3 groups (alone, in network, in group in the same clinic); presence of staff (yes or no);
4. participation and organization: number of vaccinated children, categorized as 4 groups (1–30, 31–60, 61–90, >90); number of health vaccinated children, categorized as 3 groups (1–30, 31–60, >60); number of chronic vaccinated children, categorized in 4 groups (0, 1–30, 31–60, >60); time for informed consent and registration, categorized as 4 groups (< 2 min, 2–5 min, 6–10 min, >10 min); time for vaccine execution, categorized as 4 groups (< 2 min, 2–5 min, 6–10 min, >10 min).

### Statistical analyses

To analyze the determinants of participation in the vaccination campaign we performed logistic models considering the participation in the campaign as outcome and the characteristics of the PCP as explanatory variables, adjusting by age and number of patients in care as possible confounders.

Self-reported problems were considered both individually and grouped by type (logistical, organizational and adverse reaction issues). Problems reported by non-participants are considered as possible determinants of non-participation in the vaccination campaign, while difficulties reported by participants are instead considered as issues observed during the campaign.

To analyze the level of satisfaction we only considered answers by PCPs who joined the campaign. We performed a bivariate analysis in which we described the relationship between PCPs’ level of satisfaction (variable with 5 ordered categories, from 1 (very dissatisfied) to 5 (very satisfied)) and the characteristics of the PCPs, including sex, age, residence, number of patients in care, and the vaccination skills acquired during training at postgraduate school. To assess the presence of a linear trend in the level of satisfaction reported by PCPs within the levels of these variables, the Cochran-Armitage test for trend was used. For categorical variables with more than two categories, a reference category was selected for comparison with each of the other categories within the same variable.

To ensure the stability of the estimates in the models, some modalities of categorical variables were grouped by collapsing modalities with very small sample sizes. This was done for the following variables: age range, working province, municipality residents, number of patients managed, time required for informed consent and registration, and time required for vaccine administration.

Given the absence of a power calculation to define the sample size, and the absence of pre-defined minimal difference of interest for each comparison, we do not perform any formal statistical test of hypothesis. The *p*-values are reported as quantitative continuous variables.

The statistical analyses were performed using Stata 18 (StataCorp LLC, College Station, TX, USA).

## Results

### Response rate and characteristics of the sample

At the time of the survey, there were 557 PCPs in the Emilia Romagna region, of which 271 (48%) participated in the influenza vaccination campaign. A total of 319 PCPs (57.2%) responded to the survey, of whom 223 (70.8%) participated in the campaign. The characteristics of the survey respondents and their working methods are summarized in Table 1.

**Table 1:** Demographic and professional information of PCPs responding to the survey.

		*N* (%)
Sex		
	Male	54 (17%)
	Female	264 (83%)
Age range		
	25–35	17 (5.4%)
	36–45	79 (24.8%)
	46–55	75 (23.6%)
	> 56	147 (46.2%)
Working province		
	Piacenza	24 (7.5%)
	Parma	21 (6.6%)
	Reggio Emilia	67 (21.0%)
	Modena	84 (26.3%)
	Bologna	57 (17.9%)
	Ferrara	230 (6.3%)
	Forlì-Cesena	27 (8.5%)
	Rimini	8 (2.5%)
	Ravenna	11 (3.5%)
Municipality residents		
	< 20,000	117 (38.1%)
	20,000–50,000	64 (24.1%)
	> 50,000	116 (37.8%)
Number of patients		
	< 500	14 (4.4%)
	500–700	41 (12.9)
	700–900	152 (47.8%)
	> 900	111 (34.9%)
Work organization		
	alone	68 (21.3%)
	in association	139 (43.6%)
	in group	112 (35.1%)
PCP vaccination		
	No	84 (27.5%)
	Yes	222 (72.5%)
Vaccination campaign		
	No	92 (29.2%)
	Yes	223 (70.8%)

Absolute and relative frequencies are reported for each variable.

### Determinants of participation in the campaign

We performed logistic models with participation in the campaign as outcome. The Odds Ratios are to be considered compared to the reference category for each independent variable. Except for the model which considers only PCP’s age and number of patients in care as covariates, they are all adjusted by these two variables considered as continuous, since they were linearly associated with the outcome.

Age, training about anti-flu vaccination in primary care at postgraduate school and self-reported competence in pediatric vaccinations showed a negative association with the participation in the campaign, with ORs 0.70 (95% CI 0.51–0.95), 0.54 (95% CI 0.30–0.95), and 0.62 (95% CI 0.38–1.02), respectively.

The number of patients in care, working in group clinic vs. single PCP clinic, presence of previous or current experience as Asl vaccinator and PCP’s anti-flu vaccination in 23/24 showed a strong positive association with the participation to the campaign, with ORs 1.44 (95% CI 0.99–2.10), 2.45 (95% CI 1.21–4.97), 3.12 (95% CI 1.87–5.21), and 1.94 (95% CI 1.11–3.39), respectively (Table 2).

**Table 2:** Logistic regression models showing the associations between PCP’s characteristics and participation in the vaccination campaign.

			*N*	Participants (%)	Non participants (%)	OR	95% CI
Age*						0.70	0.51–0.95
Number of patients in care**						1.44	0.99–2.10
Work organization							
Alone			68	44 (64.7)	24 (35.3)	1 (ref)	
In association			136	48 (64.7)	88 (35.3)	0.97	0.52–1.81
In group			111	91 (82.0)	20 (18.0)	2.45	1.21–4.97
Actual / past experience as ASL vaccinator							
No			131	75 (57.2)	56 (42.8)	1 (ref)	
Yes			183	148 (80.9)	35 (19.1)	3.12	1.87–5.21
Training at postgraduate school							
No			245	180 (73.5)	65 (26.5)	1 (ref)	
Yes			69	42 (60.9)	27 (39.1)	0.54	0.30–0.95
Self-reported competence in pediatric vaccination							
No			179	134 (74.9)	45 (25.1)	1 (ref)	
Yes			135	88 (65.2)	47 (34.8)	0.62	0.38–1.02
Refresh courses							
No			130	89 (68.5)	41 (31.5)	1 (ref)	
Yes			185	134 (72.4)	51 (27.6)	1.24	0.75–2.05
PCP vaccination 23/24							
No			84	54 (64.3)	30 (35.7)	1 (ref)	
Yes			219	168 (76.7)	51 (23.3)	1.94	1.11–3.39

*OR is not adjusted and is expressed per 10-years increase.

**OR is not adjusted and is expressed per 200-patients increase.

ORs are adjusted by age and number of patients in care considered as continuous. ORs are to be considered as compared to the reference category for each variable: “Work alone” for the work organization, “No” for current or previous experience as Asl vaccinator, training about anti-flu vaccination in primary care at postgraduate school, competence in pediatric vaccinations, participation in refresher courses and anti-flu vaccination 23/24 of the PCP.

### Perceived difficulties to participate in non-participants

Problems reported by non-participants are considered as possible determinants of non-participation in the vaccination campaign. 92 PCPs didn’t join the vaccination campaign, of which 23 (25% of non-participants) reported at least one difficulty. The remaining 69 (75% of non-participants), however, did not provide any response to the question about the difficulties.

**Fig. 1:**
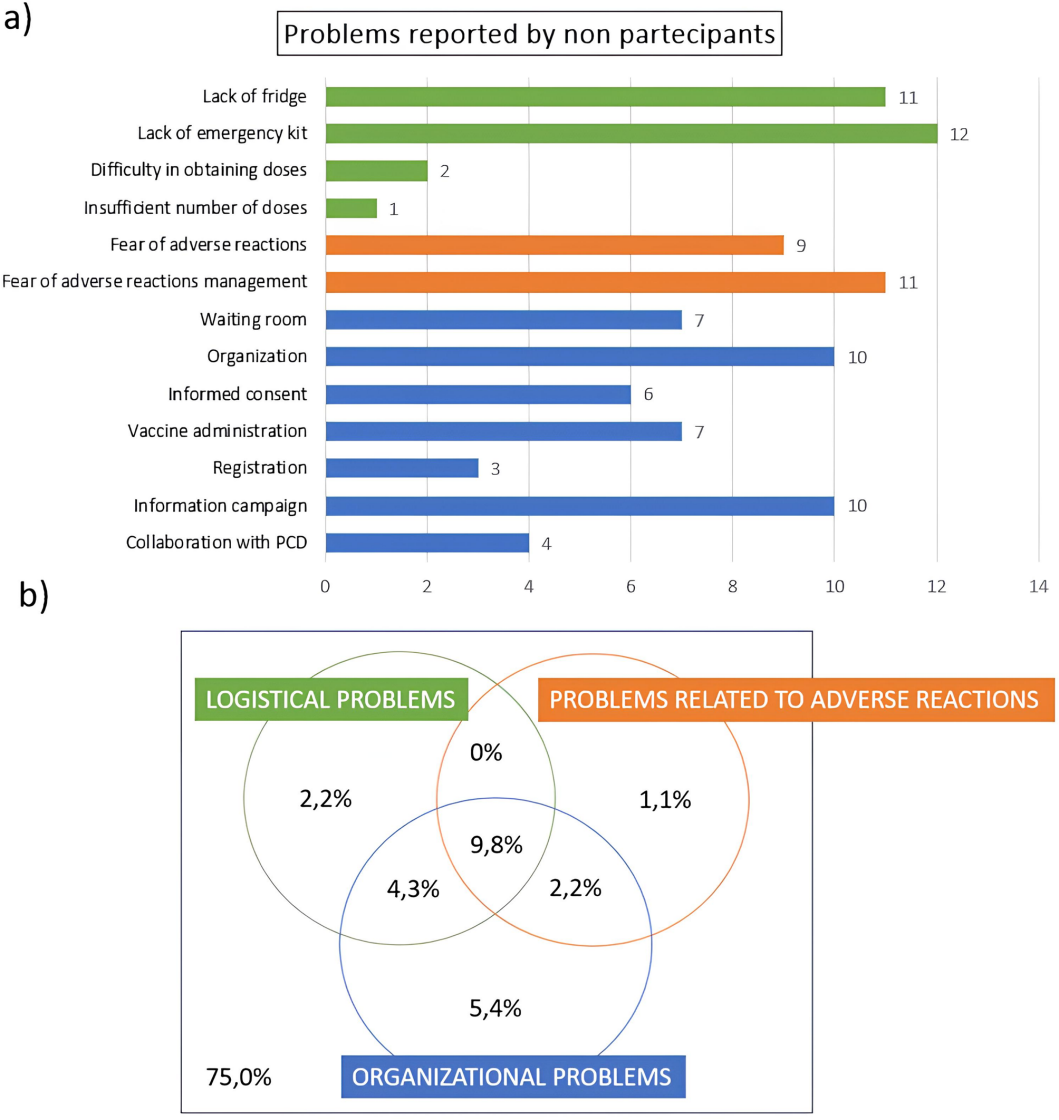
Problems reported by non-participants in the vaccination campaign: (**a**) Bar graph showing absolute frequencies of problems reported by non-participants in the vaccination campaign; (**b**) Eulero-Venn diagrams showing the percentages of those who did not report any problems, those who reported at least one logistical problem, at least one organizational problem and at least one problem concerning adverse reactions and intersections between these groups, for non-participants to the vaccination campaign.

The most reported problems concern lack of fridge (11) and emergency kit (12), organization (10), fear of managing adverse reactions (11) and low information campaign (10) (Fig. 1a).

Among those who reported at least one difficulty (25%), 9.8% reported at least one problem for each group (Fig. 1b).

### Campaign implementation and characteristics

223 PCPs declared they had joined the vaccination campaign. The volumes of activity and implementation modalities of the influenza vaccination campaign are illustrated in Table 3.

**Table 3:** Volumes of activity and implementation modalities of the influenza vaccination campaign in participating PCPs.

			*N* (%)
Number of vaccinated children			
	1–30		121 (55.0)
	31–60		54 (24.6)
	61–90		22 (10.0)
	> 90		23 (10.4)
Number of health vaccinated			
	1–30		172 (79.3)
	31–60		28 (12.9)
	> 60		17 (7.8)
Number of chronic vaccinated			
	0		97 (43.7)
	1–30		86 (38.7)
	31–60		26 (11.7)
	> 60		13 (5.9)
Place of vaccine administration			
Vaccination at office*			184 (82.5)
Vaccination in ASL*			52 (23.3)
Vaccination in other places*			6 (2.7)
Presence of personnel			
	No		118 (53.1)
	Yes		104 (46.9)
PCP vaccination			
	No		54 (24.3)
	Yes		168 (75.7)
Time for consent and registration			
	< 2 min		25 (11.5)
	2–5 min		99 (45.6)
	6–10 min		76 (35)
	> 10 min		17 (7.8)
Vaccine execution time			
	< 2 min		84 (38.5)
	2–5 min		96 (44.0)
	6–10 min		30 (13.8)
	> 10 min		8 (3.7)

Absolute and relative frequencies are reported for each variable.

*The sum of the three modalities does not necessarily correspond to the total sample since one PCP could vaccinate in more than one place

### Difficulties perceived by participants in the campaign

**Fig. 2:**
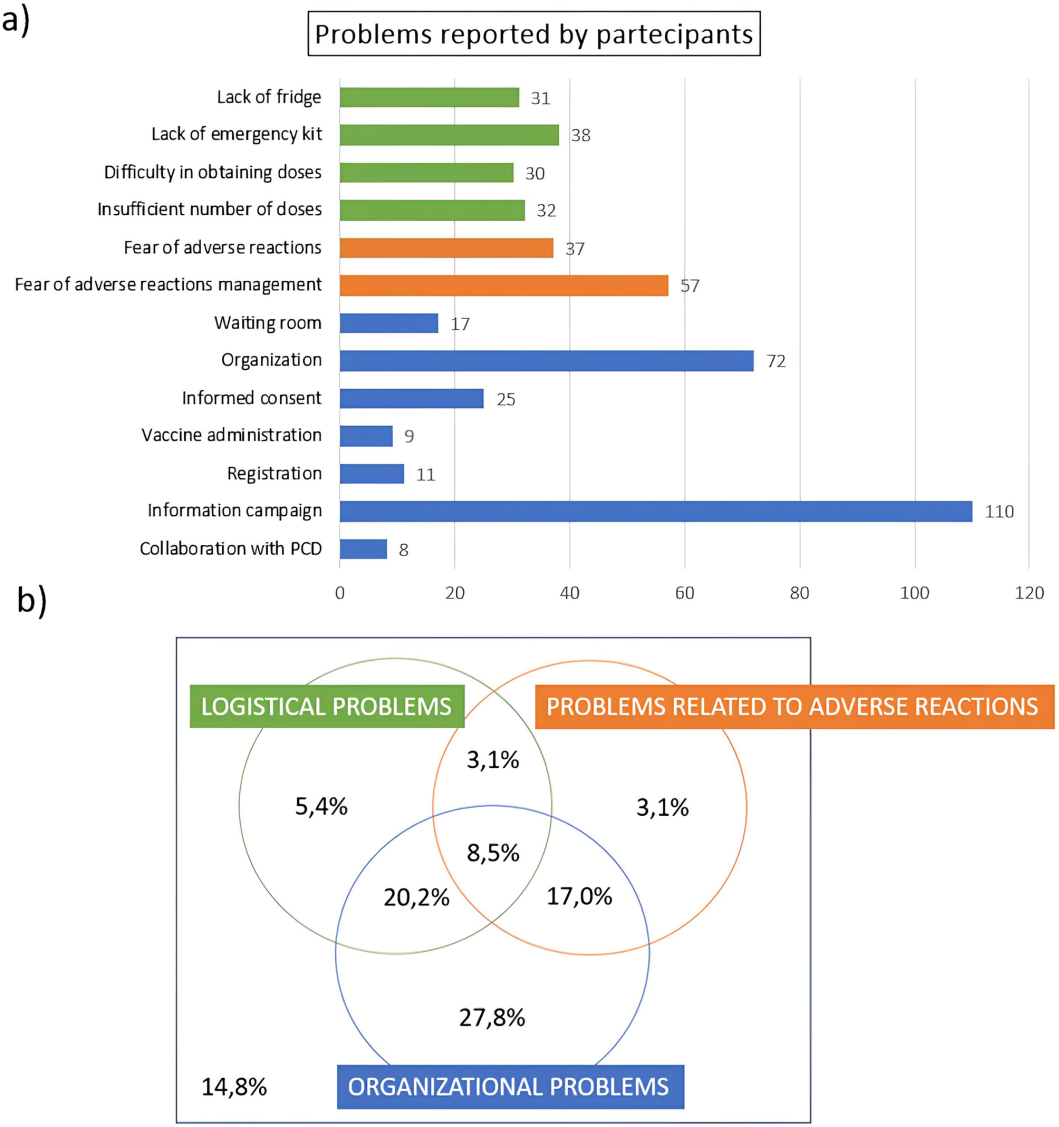
Problems reported by non-participants in the vaccination campaign: (**a**) Bar graph showing absolute frequencies of problems reported by participants in the vaccination campaign; (**b**) Eulero-Venn diagrams showing the percentages of those who did not report any problems, those who reported at least one logistical problem, at least one organizational problem and at least one problem concerning adverse reactions and intersections between these groups, for participants to the vaccination campaign.

Out of 223 PCPs who joined the vaccination campaign, 190 (85.2% of participants) reported at least one difficulty. Among the difficulties, fear of managing adverse reactions (57), organization (72) and low information campaigns (110) were reported very frequently (Fig. 2a). Difficulties encountered have been grouped as before. Among those who reported at least one difficulty (85.2%), 8.5% reported at least one problem for each group (Fig. 2b).

### Analysis of level of satisfaction

The results of the Cochran-Armitage test for trend between PCP’s characteristics and their satisfaction level are reported in Table 4. The level of satisfaction among PCPs varies based on different professional and organizational characteristics. Men report a higher level of satisfaction compared to women and PCPs with current or past experience as ASL vaccinators tend to show greater satisfaction.

**Table 4:** Mean score and distribution of satisfaction level by PCP’s characteristics.

	Mean	N (%)					P-value
		Very dissatisfied	Dissatisfied	Neither satisfied nor dissatisfied	Satisfied	Very satisfied	
Age range							
25–45	3.58	10 (5.6)	12 (13.9)	32 (16.7)	14 (44.4)	72 (19.4)	
46–55	3.71	4 (7.3)	2 (3.6)	12 (21.8)	25 (45.5)	12 (21.8)	0.523
> 56	3.77	3 (3.2)	11 (11.7)	15 (16.0)	41 (43.6)	24 (25.5)	0.283
Sex							
Female	3.64	0 (0.0)	3 (7.7)	6 (15.4)	20 (51.3)	10 (25.6)	0.102
Male	3.95	11 (6.1)	20 (11.0)	33 (18.2)	77 (42.5)	40 (22.1)	
Working province							
Emilia	3.67	10 (5.1)	22 (11.1)	38 (19.2)	82 (41.4)	46 (23.2)	0.302
Romagna	3.91	1 (4.4)	1 (4.4)	1 (4.4)	16 (69.6)	4 (17.4)	
Municipality residents							
< 20000	3.72	2 (2.4)	9 (10.8)	15 (18.1)	41 (49.4)	16 (19.3)	
20000–50000	3.64	4 (8.5)	3 (6.4)	8 (17.0)	23 (48.9)	9 (19.1)	0.653
> 50000	3.75	5 (5.9)	8 (9.4)	14 (16.5)	34 (40.0)	24 (28.2)	0.854
Number of patients							
> 900	3.85	4 (5.0)	8 (10.0)	10 (12.5)	32 (40.0)	26 (32.5)	
700–900	3.59	5 (4.7)	12 (11.3)	21 (19.8)	51 (48.1)	17 (16.0)	0.111
< 700	3.62	2 (5.9)	3 (8.8)	8 (23.5)	14 (41.2)	7 (20.6)	0.312
Work organization							
In group	3.76	5 (5.5)	8 (8.8)	13 (14.3)	43 (47.3)	22 (24.2)	
Alone	3.61	3 (6.8)	4 (9.1)	9 (20.5)	19 (43.2)	9 (20.5)	0.472
In association	3.66	3 (3.5)	11 (12.8)	17 (19.8)	36 (41.9)	19 (22.1)	0.555
Actual / past experience as ASL vaccinator							
No	3.51	3 (4.1)	12 (16.2)	18 (24.3)	26 (35.1)	15 (20.3)	0.082
Yes	3.78	8 (5.4)	11 (7.5)	21 (14.3)	72 (49.0)	35 (23.8)	
Training at postgraduate school							
No	3.73	7 (3.9)	21 (11.8)	26 (14.6)	83 (46.6)	41 (23.0)	0.325
Yes	3.55	4 (9.5)	2 (4.8)	12 (28.6)	15 (35.7)	9 (21.4)	
Self-perceived competence in pediatric vaccination							
No	3.75	6 (4.5)	13 (9.8)	22 (16.5)	59 (44.4)	33 (24.8)	0.417
Yes	3.63	4 (4.6)	10 (11.5)	17 (19.5)	39 (44.8)	17 (19.5)	
Refresh courses							
No	3.63	6 (6.8)	11 (12.5)	11 (12.5)	42 (47.7)	18 (20.5)	0.452
Yes	3.74	5 (3.8)	12 (9.0)	28 (21.0)	56 (42.1)	32 (24.1)	
Number of vaccinated children							
1–30	3.5	8 (6.7)	13 (9.2)	28 (23.5)	52 (43.7)	18 (15.1)	
31–60	3.89	1 (1.9)	5 (9.3)	6 (11.1)	29 (53.7)	13 (24.1)	0.023
61–90	4.09	1 (45.6)	2 (9.1)	2 (9.1)	6 (27.3)	11 (50.0)	0.022
> 90	3.91	1 (4.4)	2 (8.7)	3 (13.0)	9 (39.1)	8 (34.8)	0.095
Personnel							
No	3.75	4 (3.9)	11 (10.8)	18 (17.7)	43 (42.1)	26 (25.5)	0.564
Yes	3.66	7 (5.9)	11 (9.3)	21 (17.8)	55 (46.6)	24 (20.3)	
Time for informed consent and registration							
> 6 min	3.54	3 (3.3)	15 (16.3)	19 (20.7)	39 (42.4)	16 (17.4)	
2–5 min	3.79	5 (5.1)	7 (7.1)	15 (15.2)	48 (48.5)	24 (24.2)	0.097
< 2 min	3.88	3 (12.0)	1 (4.0)	1 (4.0)	11 (44.0)	9 (36.0)	0.181
Vaccine execution time							
> 6 min	3.35	2 (5.4)	8 (21.6)	8 (21.6)	13 (35.1)	6 (16.2)	
2–5 min	3.6	5 (5.21)	13 (13.5)	18 (18.6)	39 (40.6)	21 (21.9)	0.249
< 2 min	3.98	4 (4.8)	2 (2.4)	9 (10.7)	46 (54.8)	23 (27.4)	0.003
PCP vaccination 23/24							
No	3.34	6 (11.3)	7 (13.2)	12 (22.6)	19 (35.9)	9 (17.0)	0.007
Yes	3.8	5 (3.0)	16 (9.6)	27 (16.2)	78 (46.7)	41 (24.6)	

Satisfaction levels range from 1 (very dissatisfied) to 5 (very satisfied). *P*-values are computed with Cochran-Armitage test for trend between characteristics of PCPs participating in the vaccination campaign and satisfaction level. For categorical variables with more than two categories, a reference category was selected for comparison with each of the other categories within the same variable: “25–45” for the age range, “< 20000” for the number of residents in the working province, “>900” for the number of patients, “In group” for work organization, “1–30” for the number of vaccinated children, and “>6 min” for both the time required for informed consent and registration and the time for vaccine execution.

PCPs who have vaccinated a higher number of children (31–60 and 61–90) report greater satisfaction than those who have vaccinated 1–30. Similarly, shorter vaccine administration times (less than 2 min) are associated to higher satisfaction compared to times exceeding 6 min. Also, PCPs who joined the 2023/24 vaccination campaign show higher satisfaction levels than those who did not.

### Reasons for dissatisfaction

Of the 223 PCPs who participated in the vaccination campaign, 34 reported being dissatisfied or very dissatisfied. Table 5 shows the reasons for dissatisfaction indicated by the PCPs who reported being dissatisfied or very dissatisfied.

**Table 5:** Reasons for dissatisfaction among PCPs dissatisfied of the campaign.

		*N* (%)		
Organization time		16	(47.1)	
No economic advantage		15	(44.1)	
Lack of parental information		15	(44.1)	
Parental distrust		9	(26.5)	
No impact on the epidemic		6	(17.6)	
Disbelief in universal vaccination		5	(14.7)	
Difficulties in collaboration with PDC		2	(5.9)	
Limited vaccine availability		1	(2.9)	

Absolute and relative frequencies are shown. The denominator includes the 34 PCPs who reported being dissatisfied or very dissatisfied, and the numerator includes those reporting the reason for dissatisfaction who are dissatisfied or very dissatisfied.

## Discussion

The online questionnaire was completed by 57.2% of PCPs. These data far exceed those found in the literature for validated surveys on the same topic, where participation rates are typically around 20% [21, 22]. Furthermore, also part of the pediatricians who did not join the campaign responded to the survey.

Influenza vaccination coverage in children under 6 years of age and among risk groups is very low in Emilia Romagna. PCPs from Emilia responded more than those from Romagna (Rimini, Ravenna, Forlì-Cesena), where participation in the vaccination campaign was also lower (Table 1). Furthermore, in Romagna, vaccination coverage is lower for all vaccine and there are active anti-vax associations [19].

Our study found that the adherence of PCPs to the influenza vaccination campaign is mainly influenced by their previous experience in vaccine campaign with the NHS and being vaccinated themselves. There was a strong positive association between participation in the vaccination campaign and working associated within a group of PCPs. In contrast, a negative association was observed between participation in the vaccination campaign and training on influenza vaccination in primary care during postgraduate studies, as well as competence in pediatric vaccinations. However, the confidence intervals are quite wide and the difference in participation between the groups could be due to chance.

PCPs with current or past experience in vaccine campaign with the NHS are generally more satisfied, as are those who received influenza vaccination for season 23/24. PCPs who reported performance problems were on average less satisfied than those who did not. The PCPs who participated in the vaccination campaign were more satisfied with the usefulness of the vaccination than with Pay for Performance (P4P).

As expected, response rate was higher in PCPs who participated in the campaign. Furthermore, PCPs who responded to the questionnaire were more likely to work in groups or associations compared to those working alone. Among the respondents, 72.5% had been vaccinated, which far exceeds the percentage of healthcare workers who get vaccinated against influenza, which stands at 30% in Europe and 20% in Italy [1, 22], but also of the pediatricians in Italy that showed a 63% [15, 23, 24]. Being vaccinated was also associated with participating in the vaccination campaign, with 76.7% and 64.3% of participants in vaccinated and non-vaccinated pediatricians, respectively. This is consistent with previous literature [23, 24, 25], nevertheless, it is worth noting that a large proportion of PCPs not participating in the campaign is vaccinated, suggesting that even when the attitude toward flu vaccine is positive, there are other barriers to actively vaccinate children.

Factors such as the number of children in list, working in group, and previous or current experience in vaccine administration, showed a positive association with participation in the campaign. Working in group can increase adherence because many colleagues help each other during vaccination sessions and organize patient appointments for the same day. While one colleague administers the vaccine, another records the information, optimizing time and reducing the duration of vaccination sessions. Additionally, working in groups allows for larger spaces, shared medical equipment, more support staff (secretaries, nurses), and longer clinic hours. Although our survey found that the presence of support staff (secretary, nurse) did not affect the level of satisfaction of PCPs, and many of them did not use staff for the organization of the vaccination campaign. This may be due to the fact that being the first experience and having few doses available the doctor wanted to manage in the first person calls and the proposal of vaccination, to choose the patients to vaccinate. It is worth asking whether a greater availability of vaccines and longer time to organize would increase the need of nursing staff, as observed in another study [25, 11, 26]. It is worth noting that satisfaction levels increased with the increase of the number of children vaccinated and decreased with the increase of time taken for single vaccine administration. The questionnaire was not designed to collect information on the mode of vaccine administration (e.g., nasal spray vs. injection), thus, this potentially relevant factor on the organizational impact of the campaign on pediatrician clinical practice could not be evaluated in the present analysis.

Postgraduate training was inversely associated with PCPs’ participation in the campaign. It is possible that the postgraduate training offered to PCPs in our setting was more effective in raising concerns about safety and the issues in managing adverse reactions than in increasing confidence in administering vaccines and awareness of vaccination benefits.

The most common concerns about vaccinating in their clinics were similar for those who did and did not participate in the vaccination campaign: organizational challenges, fear of managing adverse reactions, and inadequate information campaigns. Among the PCPs who did not participate also emerged the lack of a fridge and emergency kit. These data confirm the three main areas of concern: logistic barriers, management of adverse reactions, and perceived lack of information by the parents. These data are in line with other studies [25, 6].

The level of satisfaction among PCPs who participated in the campaign was relatively high (66.9% reported being satisfied or very satisfied), 83.9% of PCPs stated they would repeat the experience, and 86.2% believe that influenza vaccination for healthy children can help reduce the spread of infection and lessen the burden of serious illnesses. The satisfaction level remained high across logistical challenges, concerns about adverse reactions, the PCP’s age and gender, the number of patients, and the type of work organization. However, this satisfaction was not due to the money received for the service, which was considered unsatisfactory due to low compensation. Instead, satisfaction stemmed from the perceived usefulness of the campaign. In line with previous studies [27].

Our study focused on the organizational issues that are typical of the Emilia-Romagna campaign. This approach allowed us to assess the impact of specific factors as the number of doses, the interaction with communication campaign, or the role of administrative staff. On the contrary, focusing on a given campaign did not allow to compare how different organizational models could impact on the success of the intervention. Future studies at the national and European levels could investigate differences in acceptability and implementation, by comparing the organizational characteristics of different vaccination campaigns.

### Implication for practice

#### Training

Our survey showed that postgraduate training per se does not increase the PCPs’ attitude to vaccinate. This study may contribute to better defining the contents that should be included in training or education programs. When designing such interventions, it is important to consider that younger and older workers may have different attitudes and beliefs regarding why they should take the vaccine, in fact, in our survey young PCPs more frequently participated in the vaccination campaign [16, 17, 8, 18, 28]. We identified problems in confidence in managing the adverse reactions and communication with parents. Training should focus on motivating professionals, improving their level of knowledge and counselling skills in order to address parental vaccine hesitancy, as already suggested by other authors [15, 29].

#### Organizational and logistic barriers

The number of vaccine doses given to PCPs was very limited and only in some provinces exceeded hundreds of doses, which explains why most PCPs vaccinated less than 30 children. The limited availability of vaccine doses provided by the RHS in Emilia Romagna, the insufficient public information campaign, lack of time to organize vaccination sessions, and the difficulty of managing patient lists for vaccination are organizational factors that have contributed to the low prevalence of anti-flu vaccination. The lack of guidance and training on how to manage the organization of pediatric practices made the vaccine not uniform with significant variability in the collection of informed consent, digital registration, and vaccine administration. These differences highlight the need for training aimed at improving organization and time management, which is the pediatrician’s most valuable resource, as well as proper monitoring [30]. Harmonization of the procedures and reduction of administrative burden could be more easily addressed if PCPs worked in community centers (with more appropriate spaces and vaccine storage areas). PCPs who work alone or in small groups experienced more challenges.

#### P4P

The high participation in the campaign of the PCPs in some provinces (Ferrara, Piacenza) may be partially explained by specific P4P agreements with the LHA that have been implemented only in these provinces. Nevertheless, literature indicates that P4P models are of little or dubious long-term effectiveness [31, 32, 33]. Furthermore, we noted that the economic incentives were not considered satisfying and did not determine the satisfaction. Financial incentives, which are necessary to cover the increased commitment and costs of the vaccination campaign, should be accompanied by administrative changes that ease the procedures for vaccination for both professionals and families, through changes in the administrative and logistics that should be implemented across the primary care system and not left to individual professionals [34, 35, 36, 37].

#### Information campaign

Among PCPs, the most frequently reported problem was the lack of a public information campaign for parents. The lack of an information campaign by the NHS and RHS may have hampered the participation of families, as observed in other studies [38, 39], and increased the time needed by the PCPs to inform the parents.

## Conclusions

PCPs may have a central role in increasing the anti-flu vaccination coverage in children, as shown by previous studies. Therefore, if an active role in providing vaccination is considered by the organizational model, their participation should be favored removing organizational and logistical barriers and providing effective training courses. This study indicates some critical factors on which to act to improve the practice of influenza vaccination: distribution of vaccine doses; reducing registration burden by simplifying procedures and providing administrative support; training to increase competences and confidence on how to manage adverse reactions; and increasing the level of awareness in the parents.

## Data Availability

The datasets used and analysed during the current study are available from the corresponding author on reasonable request.

## Abbreviations

LHA: Local Health Agencies
NHS: National Health System
P4P: Pay for Performance
PCD: Primary Care Doctors
PCPs: Primary care pediatricians
RHA: Regional Health Authority
