# Correction: Primary care pediatricians’ involvement in influenza vaccination campaign in Italy

**DOI:** 10.1186/s13052-025-02179-1

**Published:** 2025-12-13

**Authors:** Rosaria Indaco, Francesca Leoni, Costantino Panza, Paolo Giorgi Rossi

**Affiliations:** 1 PCPs of Azienda USL-IRCCS di Reggio Emilia, Reggio Emilia, Italy; 2 Epidemiology Unit, Azienda USL-IRCCS di Reggio Emilia, via Amendola 2, Reggio Emilia, 42122 Italy

**Correction: Italian Journal of Pediatrics (2025) 51:259**

10.1186/s13052-025-02093-6

In Table 3, the asterisk initially erroneously placed in the table description was removed and added next to the three rows “Vaccination at office”, “Vaccination in ASL”, and “Vaccination in other places”, and also in the corresponding footnote: “The sum of the three modalities does not necessarily correspond to the total sample since one PCP could vaccinate in more than one place.”

